# Supplementary material for: Public perception of COVID-19 management and response in Nigeria: a cross-sectional survey
Source: BMJ Open. 2020 Oct 14;10(10):e041936. doi: 10.1136/bmjopen-2020-041936 (PMC7559048; doi:10.1136/bmjopen-2020-041936)
Supplement: Author's manuscript [file bmjopen-2020-041936.draft_revisions.pdf]

BMJ Open is committed to open peer review. As part of this commitment we make the peer review history of every article we publish publicly available.

When an article is published we post the peer reviewers' comments and the authors' responses online. We also post the versions of the paper that were used during peer review. These are the versions that the peer review comments apply to.

The versions of the paper that follow are the versions that were submitted during the peer review process. They are not the versions of record or the final published versions. They should not be cited or distributed as the published version of this manuscript.

BMJ Open is an open access journal and the full, final, typeset and author-corrected version of record of the manuscript is available on our site with no access controls, subscription charges or pay-per-view fees (http://bmjopen.bmj.com).

If you have any questions on BMJ Open's open peer review process please email info.bmjopen@bmj.com

BMJ Open

Public Perception of COVID-19 Management and Response in Nigeria: A Cross-Sectional Survey

Journal:	*BMJ Open*
Manuscript ID	bmjopen-2020-041936
Article Type:	Original research
Date Submitted by the Author:	01-Jul-2020
Complete List of Authors:	Oleribe, Obinna; Royal Coll Physicians, International Office; Excellence and Friends Management Care Centre (EFMC), Management<br>Ezechi , Oliver; Nigerian Institute of Medical Research<br>Osita-Oleribe, Princess; Center for Family Health Initiative<br>Olawepo, Olatayo; Georgetown Centre for Public Health<br>Musa, Adesola ; Nigerian Institute of Medical Research<br>Omolabi, Anddy; Onyozar Consult<br>Fertleman, Michael; Imperial College London, Preoperative & Ageing Group<br>Salako, Babatunde; University of Ibadan<br>Taylor-Robinson, Simon; Imperial College London
Keywords:	EPIDEMIOLOGY, Organisational development < HEALTH SERVICES ADMINISTRATION & MANAGEMENT, Infection control < INFECTIOUS DISEASES

SCHOLARONE™
Manuscripts

I, the Submitting Author has the right to grant and does grant on behalf of all authors of the Work (as defined in the below author licence), an exclusive licence and/or a non-exclusive licence for contributions from authors who are: i) UK Crown employees; ii) where BMJ has agreed a CC-BY licence shall apply, and/or iii) in accordance with the terms applicable for US Federal Government officers or employees acting as part of their official duties; on a worldwide, perpetual, irrevocable, royalty-free basis to BMJ Publishing Group Ltd ("BMJ") its licensees and where the relevant Journal is co-owned by BMJ to the co-owners of the Journal, to publish the Work in this journal and any other BMJ products and to exploit all rights, as set out in our licence.

The Submitting Author accepts and understands that any supply made under these terms is made by BMJ to the Submitting Author unless you are acting as an employee on behalf of your employer or a postgraduate student of an affiliated institution which is paying any applicable article publishing charge ("APC") for Open Access articles. Where the Submitting Author wishes to make the Work available on an Open Access basis (and intends to pay the relevant APC), the terms of reuse of such Open Access shall be governed by a Creative Commons licence – details of these licences and which Creative Commons licence will apply to this Work are set out in our licence referred to above.

Other than as permitted in any relevant BMJ Author's Self Archiving Policies, I confirm this Work has not been accepted for publication elsewhere, is not being considered for publication elsewhere and does not duplicate material already published. I confirm all authors consent to publication of this Work and authorise the granting of this licence.

For peer review only

Title: Public Perception of COVID-19 Management and Response in Nigeria: A Cross-Sectional Survey

Authors: Obinna O Oleribe[1], Oliver Ezechi[2], Princess Osita-Oleribe[3], Olatayo Olawepo[4], Musa A Zaidat[2], Anddy Omolabi[5], Michael Fertleman[6], Babatunde Salako[2], Simon D. Taylor-Robinson[7].

1 – Excellence and Friends Management Care Centre (EFMC), Abuja Nigeria

2 – Nigeria Institute for Medical Research (NIMR), Lagos, Nigeria 3 –

3 – Center for Family Health Initiative (CFHI), Kubwa, Abuja Nigeria

4 – Georgetown Global Health Nigeria

5 - Onyozar Consult, Abuja, Nigeria

6 – Cutrale Perioperative & Ageing Group, Dept of Bioengineering, Imperial College London, White City Campus, 86 Wood Lane, London W12 0BZ, UK

7 - Department of Surgery and Cancer, Imperial College London, W2 1NY

Corresponding Author: Dr Michael Fertleman, Cutrale Perioperative & Ageing Group, Dept of Bioengineering, Imperial College London, White City Campus, 86 Wood Lane, London W12 0BZ, UK email: m.fertleman@imperial.ac.uk

Short title: Public Perspective of COVID-19 in Nigeria

Key words: COVID-19; Nigeria; public perception; cross-sectional survey

For peer review only - http://bmjopen.bmj.com/site/about/guidelines.xhtml

Contributorship Statement

OOO developed the concept, initial tool and first draft manuscript.
OE and POO reviewed the final tool, made inputs to the original draft manuscript, and supported data analysis.
OOO, OO, MAZ and AO did the data analysis
AO did the spatial analysis.
MF, BS and STR reviewed, edited and finalized the full manuscript.
STR provided quality assurance to the project.

Abstract

Objectives: A study designed to assess the public perception of the response of government and its institutions to the COVID-19 epidemic in Nigeria.

Setting: Self-selecting participants throughout Nigeria completed a self-administered questionnaire through an online cross-sectional survey.

Participants: 495

Results: The majority of respondents were married (76.6%), were males (61.8%), had tertiary level education (91.0%), were public servants (36.8%), Christians (82.6%), and resident either in the Federal Capital Territory (Abuja) (49.1%) or in the South-East Region of Nigeria (36.6%). Over 95% of the respondents had heard of COVID-19 (98.8%) and knew it is a viral disease (95.4%). The government and its institutions response to the pandemic were rated as poor, with the Federal President's Office having the highest level of poor rating (57.5%). Communication (50.0%) and prevention messages (43.7%) received the highest perception good rating. Female respondents and those less than 40 years generally rated the governmental responses as poor.

Conclusions/Recommendations: It is recommended that as a public-private partnership approached was efficiently used to more effectively disseminate public health communication and prevention messages, the Nigerian Government should expand this collaboration to improve the quality of services provided in other areas of COVID-19 outbreak management. In addition, the Nigerian Government and its agencies should improve their financial and human resource stewardship roles in response to COVID-19.

For peer review only - http://bmjopen.bmj.com/site/about/guidelines.xhtml

Article summary

Strengths and limitations of this study

- First African study of public perception of government response to the COVID 19 pandemic
- Study was cross sectional and questionnaire was self administered thereby reducing researcher induced bias
- Study provides perspectives from different classes of Nigerians on the COVID 19 pandemic
- While providing evidence for informed decisions by national leaders and program managers the study period was short and therefore limited
- Only individuals with social media account or internet access could participate in the study

For peer review only

For peer review only - http://bmjopen.bmj.com/site/about/guidelines.xhtml

Introduction

The world has changed since the outbreak of Coronavirus Disease 2019 (COVID-19) in Wuhan in China in December 2019 with its causative agent, the severe acute respiratory syndrome coronavirus 2 (SARS-CoV-2) identified.[1,2,3,4] As COVID-19 has no specific antiviral drug treatment or vaccine to date, universal safety precautions (USPs) and mitigating strategies are the only common way to deal with this global health emergency[4].

Telemedicine, forward triage before patients arrive at an emergency center, and social distancing measures which protect patients, clinicians, and the community from exposure and allow physicians and patients to communicate when necessary using smartphones or webcam-enabled computers have been proposed to address challenges posed by COVID-19[5]. Moreover, because the COVID-19 outbreak posed significant challenges for the public health, research and medical communities, governmental policies and processes were introduced to curtail the spread of the virus across the nations of the world[6]. This included instituting temporary restrictions on travel with dramatic reduction in the number of travelers, self distancing, self isolation, quarantine of international travellers, regular hand washing and the use of face masks[6,7,8,9,10]. These policies have their economic implications to both developed and developing nations like Nigeria[11].

In Nigeria, most of these global policies were adopted and implemented without a review of their effectiveness and implications to the sociocultural climate of the nation. For instance, social distancing measures may save many lives in high-income countries, but are less effective in poor countries with younger populations who are less susceptible to COVID-19. Such measures are associated with healthcare systemic, economic, and sociocultural challenges[11].

Poor people are less willing to make economic sacrifices, not because they place greater value on their livelihood concerns than contracting COVID-19[11], but because they lack the resources and social protections to isolate themselves and sacrifice economic opportunities[11]. The country formulated new policies, developed new guidelines and standard operating procedures (SOPs), and established several committees including the National Emergency Organizing Committee and Presidential Task Force (PTF) [12,13,14] to respond to the pandemic. The governmental efforts at all levels were complemented by

For peer review only - http://bmjopen.bmj.com/site/about/guidelines.xhtml

donations and contributions from the organised private sector, health professional associations, non-governmental organisations (NGOs) and individual volunteers [15,16,17].

However, the pandemic in Nigeria continues unabated despite the various efforts of individuals, government and organised private sector with 46,803 tested, 8,344 confirmed cases and 249 deaths in 3 months (February 27 – May 27, 2020) of the epidemic in Nigeria[18], prompting plan to introduce addition measures.

The public perception, opinion and attitude to the various social control interventions introduced in the wake of the epidemic have not previously been evaluated. Information obtained may be useful in planning, modifying and implementing a coordinated response to current and future epidemics in Nigeria. Since it is established that public perception and attitude are important for programme success, this study was designed to assess, document, and evaluate public knowledge and perception of the measures introduced to combat the COVID-19 epidemic in Nigeria.

For peer review only

For peer review only - http://bmjopen.bmj.com/site/about/guidelines.xhtml

Method

Timeline

This study was conducted between May 15, 2020 to May 21, 2020 inclusive, through a nationwide, anonymous online cross-sectional survey.

Participants

Participants were obtained using a self-selecting sampling technique. The participants were informed of the anonymity of their participation and that information collected would be kept confidential. Only participants 18 years and above were invited to participate in the survey

Sample size

We calculated an approximate minimum sample size of 452 at 5% precision, 95% confidence interval, and 50% response distribution (online survey) (http://www.raosoft.com/samplesize.html).

Patient and public involvement

Between 10-12 May 2020 the tool was developed. Between 13-15 May 2020 the tool was reviewed with seven members of the public at the lead author's institution, before finalising with their input.

No patients were involved in the design of the study.

Survey

Google Form (https://docs.google.com/forms/d/1P36vWQ0Ihf8HMTLHWIF83aZy-hFKVla7FSSm-WZudMY/edit) was used to develop and distribute the questionnaire. Between 10-12 May 2020 the tool was developed. Between 13-15 May 2020 the tool was reviewed with seven members of the public, before finalising with their input. The study was designed as an online survey to cover the entire country during the 7-day period. The questionnaire was in English and was pretested for comprehensibility, acceptability and accuracy before the study was commenced. Invitations to participate in the study were through private messages, social groups, and on several social networking sites and platform (emails, WhatsApp and Facebook). By clicking on a link, the participant was

For peer review only - http://bmjopen.bmj.com/site/about/guidelines.xhtml

directed to the survey's entry page (https://docs.google.com/forms/d/e/1FAIpQLSf4iuWGfO4pIvSVi24Sn0DhZSO9q_wnpYhB2J3boAsQ7W4Uww/viewform?vc=0&c=0&w=1&usp=mail_form_link), which contained information on the objectives of the survey, terms of participation and data privacy. Participants were also informed about the possible risks and benefits of the survey. They were asked to complete the survey in one session and that it would take between 10 to 15 minutes. Participants were able to access the survey and complete it on a computer or a mobile device.

The survey consisted of 30 semi-structured questions. The questionnaire collected information on sociodemographic characteristics, knowledge of COVID-19 and individual perspective on management and response to the pandemic. The questions were semi-structured as some questions allowed participants to provide detailed written answers to obtain a more profound sense of their perspective. The data collected for the survey and utilized in this study were stored electronically and were password protected.

Measures

Sociodemographic Characteristics: Participants responded to closed-ended demographic questions including their gender, age, relationship status, city and state of residence, work status, and educational status.

Response to COVID-19: Participant perception of the measures introduced by the Federal Government and governmental institutions to COVID-19 pandemic were assessed in the survey. The respondent perception of scores ranging from lowest score of 1 (not satisfied at all) to highest score of 10 (very satisfied) were further classified as poor (score of 1-4, average (score of 5-7) and good (scores of 8-10).

Data analysis

Data collected on Google Analytic Tool were exported to and analysed with the Statistical Package for Social Sciences for Windows version 23.0 (SPSS Inc., Chicago, IL). A p-value of <0.05 was considered statistically significant.

For peer review only - http://bmjopen.bmj.com/site/about/guidelines.xhtml

Results:

A total of 495 respondents returned completed questionnaires during the study period. While 482 (97.4%) respondents were resident in Nigeria, 13 (2.6%) were temporarily resident outside the country owing to border closures. The age of the respondents ranged from 18 to 59 years, with an average age of 42.1 years (standard deviation = 9.7). The majority of respondents were married (76.6%), males (61.8%), had tertiary level education (91.0%), were public servants (36.8%), Christians (82.6%), and resident either in the Federal Capital Territory (49.1%) or the South-East region (36.6%) (Table 1). Over 95% of the respondents had heard of COVID-19 (n = 489, 98.8%), and knew that it is a viral disease (n = 472; 95.4%).

Table 2 shows the respondent perception of the response of the Federal Government and the governmental institutions to the COVID-19 outbreak in Nigeria. The government measures were rated as poor, with the Office of the Presidency having the highest poor rating score by 57.5% of the respondents. The Federal Ministry of Health, the Presidential COVID-19 Task Force, and the Nigerian Centre for Diseases Control responses to the outbreak were rated as poor by 46.1%, 45.8% and 36.0% respectively. The respondents perception of specific governmental and institutional responses to COVID-19 are detailed in Table 3. Among the specific responses by Government and its institutions to the pandemic, public health communication about the epidemic and general prevention messages received the highest good rating by 50.0% and 43.7% of the respondents respectively. The availability of social and economic support from the Federal Government (n = 406; 82.9%) had the highest poor rating score, followed by governmental management of the resources provided for the outbreak (n = 368; 75.4%) and readiness of the government to manage the epidemic (65.5%). Social and economic support from religious bodies and family and friends were rated as poor by 52.1% and 33.1% of the respondents respectively.

The respondent perception of governmental and institutional response by respondent age group and sex is shown in table 4. The proportion of respondents less than 40 years that rated the governmental and institutional measures to combat COVID-19 as poor were greater than those 40 years and above across all aspects of the survey. However, it is

For peer review only - http://bmjopen.bmj.com/site/about/guidelines.xhtml

only in the perception of the Office of the Presidency that the difference was statistically significantly higher (p=0.03). The proportion of female respondents who rated the governmental measures as poor were higher compared to their male counterpart across all aspects of the survey. The observed difference was only statistically significantly higher again in the perception of the response of the Office of the Presidency (p=0.05).

A spatial dimension of the responses was also analysed by aggregating the ratings of the respondents by their state of residence with the results presented in figure 1. Responses were from 33 states including the Federal Capital Territory (n=33). Respondents from 10 states (30%) rated the response of government as good, while respondents from 12 states (36%) rated the governmental response as average, while the respondents from the remaining 11 states (33%) rated the responses as poor. Analyses of the rating by states, indicated that Lagos states, even though it has the highest number of laboratory confirmed COVID-19 cases in the country 3,756 (45%), rated the government response as good. This contrasts with Kano state, which comes second with 923 (11%) confirmed cases, but rated the governmental responses as poor. The FCT, which comes third in terms of confirmed cases 519 (6%), rated the government response as average. To determine the statistical significance of the relationship between the responses and the number of confirmed COVID-19 cases, the Pearson's correlation was applied. A Pearson's coefficient of 0.11 and a p value of 0.51 were obtained, indicating a weak and insignificant relationship between the responses and number of confirmed COVID-19 cases.

Data Sharing

No additional data will be available.

For peer review only - http://bmjopen.bmj.com/site/about/guidelines.xhtml

Discussion:

The success and sustainability of public health programmes are highly dependent on the positive perception and acceptance by the general public. The continued increase in new cases of COVID-19 3 months after the first case was reported in Nigeria in February 27 2020 has made Nigerians compare the governmental response to COVID-19 with that of the successful containment of Ebola Viral Disease (EVD) in 2016. It is reasonable to speculate that if the same response in terms of promptness of attention, leadership, networking and commitment given to EVD epidemic was given to COVID-19, that the number of new cases, morbidity and mortality from COVID-19 would have seen an end to the outbreak or at least a reduction in numbers affected. As a result of this failure to cap the outbreak, significant numbers of the public are disillusioned and are not keeping to the government-issued guidelines and recommendations, with some believing that the COVID-19 pandemic is a hoax. This study was conducted to evaluate the perceptions of Nigerians to the measures taken by the Federal Government and its governmental institutions in response to the COVID-19 outbreak in the country.

In this study, the majority of the respondents were resident in Nigeria, had tertiary level education, were males having heard of COVID-19 and knew its viral aetiology (95.4%). The study shows that the respondents were educated and had some level of knowledge of COVID-19, and thus could form a meaningful opinion on the governmental response. The respondents' residency in Nigeria highlights that their opinions are well informed and could also impact on how the government guidelines and recommendations might be observed by the general public. The high educational attainment of the respondents may be a reflection of their awareness of importance and the understanding of the relevance of research. The male preponderance in this study may be a reflection of previously observation phenomena that gender plays a role in participation in online surveys[19].

The study shows that the respondents were unhappy with the manner of responses provided by the Federal Government and its agencies including the Office of the Presidency, the Presidential Task Force on COVID 19 (PTF), the Federal Ministry of Health (FMOH), and the Nigerian Center for Disease Control (NCDC). Apart from NCDC, over 70% of all respondents scored the Office of the Presidency, PTF and FMOH poor or

For peer review only - http://bmjopen.bmj.com/site/about/guidelines.xhtml

average with 57% scoring the Office of the Presidency as poor in its performance. This may be an indication of either a delayed or an inadequate response to the crisis, which may have contributed to the increasing number of new cases of COVID-19 and deaths in Nigeria 3 months after the first case was reported.

Similarly, the majority of the respondents rated specific individual measures by the government and its institutions as poor. Over 50% of the respondents scored the management of isolation, and treatment/diagnostic centres as poor. This is in support of the numerous social media videos and comments against these centres across Nigeria. Communication and delivery of prevention messages accrued better scores. This specific area of public health messaging was provided through a public-private partnership between the government agencies and global system for mobile technology operators in the country. It is therefore not surprising that the public had positive perceptions relative to other specific measures as the private sectors were more efficient and effective in conveying information on COVID-19 programmes and policies.

Unfortunately, the enforcement of the implementation of the guidelines and policies are still poor. Mainly because of the negative perception of governmental measures taken, individuals have not taken the pandemic seriously. The situation is compounded by the presentation of COVID-19 in Nigeria where a significant number of cases have been asymptomatic and the case fatality rate has been low[20] making people believe that the disease is not a serious illness[21].

Most social and economic support came from family and friends, followed by the religious bodies. The majority (over 80%) believed that government social welfare provisions were poor. One cannot however, identify the root cause of this perception as funds were made available for supportive measures for sectors of the population in Nigeria. Whether the funds were not adequately released, released but diverted or poorly distributed remains to be seen. This calls for new in-depth studies to identify the root cause of this unsatisfactory aspect of governmental performance during this crisis.

Over 65% of the participants believed that the government was not prepared for the outbreak and 75% that the government poorly managed the resources provided for the outbreak. It is not therefore surprising to note that the government was asking returnees

For peer review only - http://bmjopen.bmj.com/site/about/guidelines.xhtml

to pay for their quarantine or be allowed to suffer the consequences – which may include denial of access to the country[22].

Social support, enforcement of isolation policies, and provision of testing services did not go well, according to the participants. These are project management issues that the engagement of a professional project manager to operationalize the entire process would have eliminated. It is therefore important that skilled project managers are engaged in subsequent epidemics to mitigate poor project management, better finance management, improve quality of services and to ensure timely delivery of results and benefits.

Given a large proportion of the population under the age of 40 years is active on social media, it seems prudent to recommend that governmental policy for future epidemics should use this medium for communication to a growing sector of Nigerian society.

The findings of this online cross-sectional study have to be interpreted with some limitations in mind. The use of an online survey for this study may have resulted in a selection bias for respondents who are educated, internet savvy and have a social media account. It may have limited some category of respondents from participating. Respondents without formal education, without internet connection or who live in rural areas may have been disenfranchised. However, this will not invalidate the findings presented here, more especially since only those who are knowledgeable and know the specific governmental measures taken, can meaningfully assess what is being done. Finally, this is a cross-sectional study, and does not allow any inferences to be made regarding causality and temporality with respect to perception and the socio-demographic factors.

To our knowledge, this is among the first studies to examine the perception of governmental response by the public in West Africa. Information obtained here may be useful to guide future decisions especially when considering the impact of public perception on uptake of governmental guidelines and policies. Also, for the first time in the country, we have gained information on public perception of governmental responses during a global pandemic. Finally, this study utilised nationally-representative data with appropriate sample size, which strengthens the external validity and generalizability of the study.

For peer review only

For peer review only - http://bmjopen.bmj.com/site/about/guidelines.xhtml

Conclusions

The study shows a generally poor perception of governmental response to the COVID-19 pandemic by the public. All the specific interventions against COVID-19 were rated as poor except for public health communication and prevention messages. It is recommended that the public-private partnership approached used in this particular area should be adopted in the implementation all areas of COVID-19 outbreak policy to streamline perceived and actual inefficiencies.

Acknowledgement

We are grateful to the members of the Nigerian public who helped with the survey design. We acknowledge the National Emergency Outbreak Committee on COVID 19, the Presidential Task Force (PTF) and the Nigerian Center for Disease Control. We celebrate all the frontline healthcare workers who put their lives at risk to control and contain this epidemic. We thank the EFMC and HS3 Team for their excellent work during this period. MF and SDTR are grateful to the NIHR Biomedical Facility at Imperial College London for infrastructural support. SDTR was supported by a Wellcome Trust ISSF grant at Imperial College London.

Declaration

Authors have nothing to declare.

For peer review only - http://bmjopen.bmj.com/site/about/guidelines.xhtml

References

1. Fang Y, Zhang H, Xie J, et al. Sensitivity of Chest CT for COVID-19: Comparison to RT-PCR. *Radiology*. February 2020:200432. doi:10.1148/radiol.2020200432
2. Huang C, Wang Y, Li X, et al. Clinical features of patients infected with 2019 novel coronavirus in Wuhan, China. *Lancet*. 2020;395(10223):497-506. doi:10.1016/S0140-6736(20)30183-5
3. WHO | Pneumonia of unknown cause – China. https://www.who.int/csr/don/05-january-2020-pneumonia-of-unkown-cause-china/en/. Accessed May 19, 2020.
4. Gudi SK, Undela K, Venkataraman R, et al. Knowledge and Beliefs towards Universal Safety Precautions to flatten the curve during Novel Coronavirus Disease (nCOVID-19) Pandemic among general Public in India: Explorations from a National Perspective. *medRxiv*. 2020:2020.03.31.20047126. doi:10.1101/2020.03.31.20047126
5. Hollander JE, Carr BG. Virtually Perfect? Telemedicine for Covid-19. *N Engl J Med*. March 2020. doi:10.1056/NEJMp2003539
6. Fauci AS, Lane HC, Redfield RR. Covid-19 - Navigating the uncharted. *N Engl J Med*. 2020;382(13):1268-1269. doi:10.1056/NEJMe2002387
7. World Health Organization (WHO). Rational use of personal protective equipment for coronavirus disease 2019 (COVID-19) and considerations during severe shortages. *Who*. 2020;(April):1-28. https://apps.who.int/iris/handle/10665/331695.
8. WHO. Rational use of personal protective equipment for coronavirus disease 2019 (COVID-19). *Who*. 2020;2019(February):1-7.
9. van Bavel JJ, Baicker K, Boggio PS, et al. COVID-19 pandemic response. *Nat Hum Behav*. 2020;4(5):1-12. doi:10.1038/s41562-020-0884-z
10. Raju E, Ayeb-Karlsson S. COVID-19: How do you self-isolate in a refugee camp? *Int J Public Health*. May 2020:1-3. doi:10.1007/s00038-020-01381-8
11. Barnett-Howell Z, Mobarak AM. The Benefits and Costs of Social Distancing in Rich

For peer review only - http://bmjopen.bmj.com/site/about/guidelines.xhtml

and Poor Countries. April 2020. http://arxiv.org/abs/2004.04867. Accessed May 19, 2020.

12. NCDC Coronavirus COVID-19 Microsite. https://covid19.ncdc.gov.ng/resource/. Accessed May 21, 2020.

13. NCDC Coronavirus COVID-19 Microsite. https://covid19.ncdc.gov.ng/guideline/. Accessed May 21, 2020.

14. Presidential Task Force on Power. https://statehouse.gov.ng/covid19/. Published 2015. Accessed May 19, 2020.

15. Nigerian private sector donates more than most other African countries in fight against COVID-19 - African Business Magazine. https://africanbusinessmagazine.com/sectors/health-sectors/covid-19/nigerian-private-sector-donates-more-than-most-other-african-countries-in-fight-against-covid-19/. Accessed May 19, 2020.

16. Did Nigeria Hit the 100 Billion Mark in COVID-19 Donations?https://www.premiumtimesng.com/coronavirus/388065-did-nigeria-hit-the-100-billion-mark-in-covid-19-donations.html. Accessed May 19, 2020.

17. COVID-19: Lagos commissions 118-bed isolation centre.https://www.premiumtimesng.com/regional/ssouth-west/390886-covid-19-lagos-commissions-118-bed-isolation-centre.html. Accessed May 19, 2020.

18. NCDC Coronavirus COVID-19 Microsite. https://covid19.ncdc.gov.ng/. Accessed May 22, 2020.

19. Bowale A, Abayomi A, Idris J, et al. Clinical presentation, case management and outcomes for the first 32 COVID-19 patients in Nigeria. *Pamj*. 2020;35(Supp 2):1-4. doi:10.11604/PAMJ.SUPP.2020.35.2.23262

20. Smith G, Smith WG. *Does Gender Influence Online Survey Participation?: A Record-Linkage Analysis of University Faculty Online Survey Response Behavior.*; 2008. https://scholarworks.sjsu.edu/elementary_ed_pub. Accessed June 8, 2020.

For peer review only - http://bmjopen.bmj.com/site/about/guidelines.xhtml

21. Unicef warns lockdown could kill more than Covid-19 as model predicts 1.2 million child deaths. https://www.telegraph.co.uk/global-health/science-and-disease/unicef-warns-lockdown-could-kill-covid-19-model-predicts-12/. Accessed June 8, 2020.

22. Covid-19: Nigerian evacuees from Thailand must pay N297,600 for quarantine, feeding -FG - Businessday NG. https://businessday.ng/coronavirus/article/covid-19-nigerian-evacuees-from-thailand-must-pay-n297600-for-quarantine-feeding-fg/. Accessed June 8, 2020.

For peer review only

For peer review only - http://bmjopen.bmj.com/site/about/guidelines.xhtml

Table 1: Sociodemographic characteristics of respondents in the study

Characteristics	Number of respondents (%)
**Country of Residence**	
Nigeria	482(97.4)
Others	13(2.6)
**Geopolitical zone of Residence**	
**North-Central**	243(49.1)
**North-East**	29(5.9)
**North-West**	30(6.1)
**South-East**	68(13.7)
**South-South**	39(7.9)
**South-West**	86(17.4)
**Marital Status**	
Single	100(20.2)
Married	379(76.6)
Divorced/Separated	9(1.8)
Widowed	7(1.4)
**Age (years)**	
Less than 20	1(0.2)
20 - 29	61(12.3)
30 - 39	142(28.7)
40 - 49	159(32.1)
50 - 59	93(18.8)
60 years and above	39(7.9)
**Sex**	
Female	189(38.2)
Male	306(61.8)
**Religion**	
African Traditional Religion	2(0.4)
Christianity	409(82.6)
Islam	79(16.0)
Others	3(0.6)
**Education qualification**	
Non formal	18(3.6)
Primary	2(0.4)
Secondary	20(4.0)
Tertiary	455(91.9)
**Work status**	
Working	338(87.5)
Not working	38(7.7)
Student	24(4.8)

For peer review only

For peer review only - http://bmjopen.bmj.com/site/about/guidelines.xhtml

Table 2: Respondent perception of the Governmental and Governmental Institutional response to the COVID 19 pandemic in Nigeria

Government /Institution	Respondents COVID -19 response perception rating		
	Poor n (%)	Average n (%)	Good n (%)
The Presidency (N=487)	280(57.5)	130(26.7)	77(15.8)
Presidential Task Force on COVID-19 (N=491)	225(45.8)	168(34.2)	98(20.0)
Federal Ministry of Health (FMOH) (N=490)	226(46.1)	164(33.5)	490(20.4)
Nigerian Center for Disease Control (NCDC) (N=492)	177(36.0)	157(31.9)	158(32.1)

For peer review only

For peer review only - http://bmjopen.bmj.com/site/about/guidelines.xhtml

Table 3: Respondents perception of the Governmental and Governmental Institutional specific response to COVID-19 outbreak in Nigeria

Specific response	Respondents COVID -19 response perception rating		
	Poor n (%)	Average n (%)	Good n(%)
Enforcement of Stay at home, social distancing, face mask, and handwashing policies (N=492)	244(49.6)	141(28.7)	107(21.7)
Management of Isolation Centres (N=482)	265(55.0)	148(30.7)	69(14.3)
Management of treatment and diagnostic centres (N=477)	254(53.2)	150(31.4)	73(15.3)
Communication and information about the epidemic (N=490)	129(26.3)	116(23.7)	245(50.0)
Prevention messages from government (N=490)	146(29.8)	130(26.5)	214(43.7)
Use of Personal Protective Equipment (PPE) by healthcare workers (N=484)	232(47.9)	134(27.7)	118(24.4)
Availability of social and economic support from Government (N=490)	406(82.9)	53(10.8)	31(6.3)
Availability of social and economic support from Religious bodies (N=486)	253(52.1)	129(26.5)	104(21.4)
Availability of social and economic support from family and friends (N=487)	161(33.1)	158(32.4)	168(34.5)
The readiness of the government to manage the epidemic (N=490)	321(65.5)	102(20.8)	67(13.7)
Government management of the resources provided for the outbreak (N=488)	368(75.4)	82(16.8)	38(7.8)

For peer review only

For peer review only - http://bmjopen.bmj.com/site/about/guidelines.xhtml

Table 4: The Respondents perception of Government and Institution response to COVID 19 pandemic in Nigeria by Sex and Age-group

Government/Institution	Respondents COVID -19 response perception rating						P-value
	Poor	Average	Good	Poor	Average	Good	
	**Male**			**Female**			
**The Presidency (N=487)**	167(59.6)	80(61.5)	56(72.7	113(40.4)	50(38.5)	21(27.3	0.05
**Presidential Task Force on COVID-19 (N=491)**	130(57.8)	112(66.7)	62(63.3)	95(42.2)	56(33.3)	36(36.7)	0.19
**Federal Ministry of Health (FMOH) (N=490)**	128(56.6)	115(70.1)	60(60.0)	98(43.4)	49(29.9)	40(40.0)	0.23
**Nigerian Center for Disease Control (NCDC) (N=492)**	104(58.8)	102(65.0)	99(62.7)	73(41.2)	55(35.0)	59(37.3)	0.44
	**Less than 40 years**			**40 years and above**			
**The Presidency (N=487)**	126(45.0)	51(39.2)	25(32.5)	154(55.0)	79(60.8)	56(72.7)	0.03
**Presidential Task Force on COVID-19 (N=491)**	98(43.6)	70(41.7)	35(35.7)	127(56.4)	98(58.3)	63(64.3)	0.21
**Federal Ministry of Health (FMOH) (N=490)**	92(40.7)	73(44.5)	38(38)	134(59.3)	61(55.5)	62(62.0)	0.83
**Nigerian Center for Disease Control (NCDC) (N=492)**	68(78.4)	70(44.6)	66(41.8)	109(61.6)	87(55.4)	92(58.2)	0.51

For peer review only - http://bmjopen.bmj.com/site/about/guidelines.xhtml

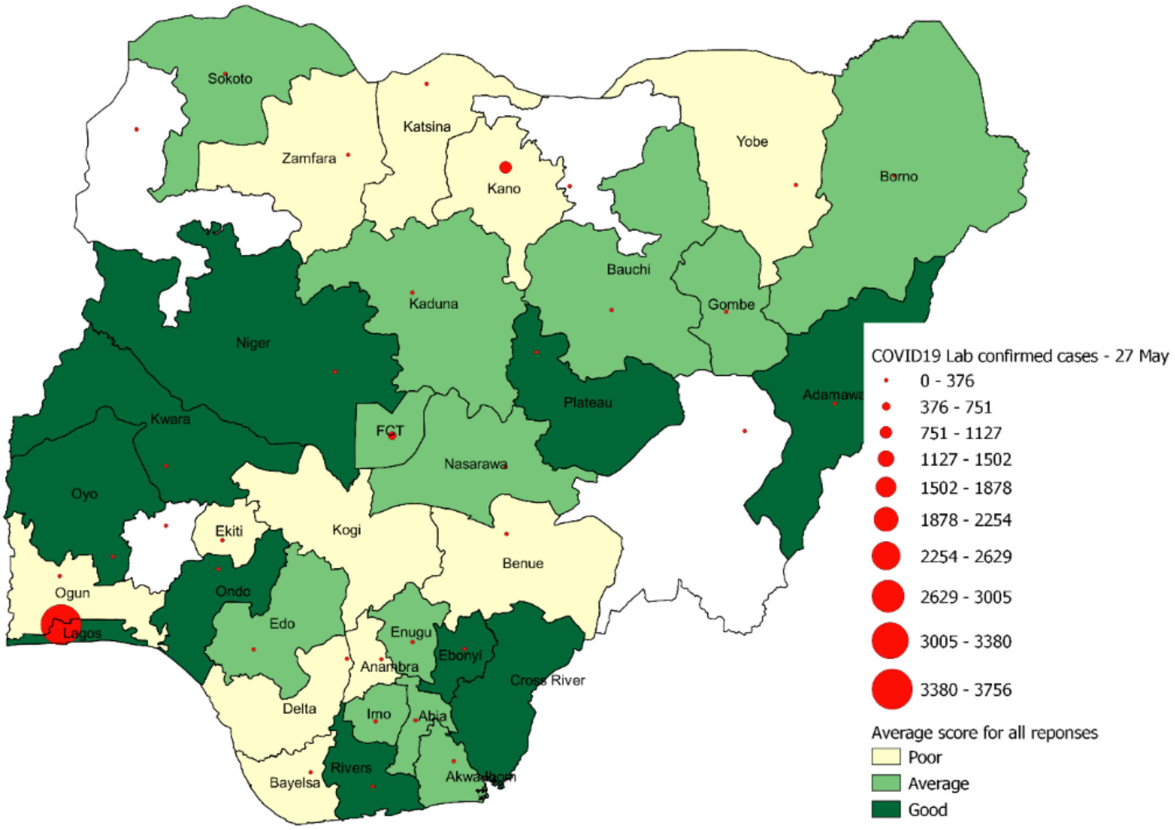
Sokoto
Katsina
Zamfara
Kano
Yobe
Borno
Bauchi
Gombe
Kaduna
Niger
Kwara
FCT
Plateau
Nasarawa
Adamawa
Oyo
Ekiti
Kogi
Benue
Ogun
Ondo
Lagos
Edo
Enugu
Anambra
Ebonyi
Cross River
Delta
Imo
Abia
Rivers
Bayelsa
Akwa Ibom
COVID19 Lab confirmed cases - 27 May
0 - 376
376 - 751
751 - 1127
1127 - 1502
1502 - 1878
1878 - 2254
2254 - 2629
2629 - 3005
3005 - 3380
3380 - 3756
Average score for all reponses
Poor
Average
Good

review only

Public Perception of COVID-19 Management and Response in Nigeria: A Cross-Sectional Survey

Journal:	*BMJ Open*
Manuscript ID	bmjopen-2020-041936.R1
Article Type:	Original research
Date Submitted by the Author:	24-Aug-2020
Complete List of Authors:	Oleribe, Obinna; Royal Coll Physicians, International Office; Excellence and Friends Management Care Centre (EFMC), Management<br>Ezechi , Oliver; Nigerian Institute of Medical Research<br>Osita-Oleribe, Princess; Center for Family Health Initiative<br>Olawepo, Olatayo; Georgetown Centre for Public Health<br>Musa, Adesola ; Nigerian Institute of Medical Research<br>Omolabi, Anddy; Onyozar Consult,<br>Fertleman, Michael; Imperial College London, Preoperative & Ageing Group<br>Salako, Babatunde; University of Ibadan<br>Taylor-Robinson, Simon; Imperial College London
**Primary Subject Heading**:	Public health
Secondary Subject Heading:	Epidemiology, Health policy, Infectious diseases
Keywords:	EPIDEMIOLOGY, Organisational development < HEALTH SERVICES ADMINISTRATION & MANAGEMENT, Infection control < INFECTIOUS DISEASES

I, the Submitting Author has the right to grant and does grant on behalf of all authors of the Work (as defined in the below author licence), an exclusive licence and/or a non-exclusive licence for contributions from authors who are: i) UK Crown employees; ii) where BMJ has agreed a CC-BY licence shall apply, and/or iii) in accordance with the terms applicable for US Federal Government officers or employees acting as part of their official duties; on a worldwide, perpetual, irrevocable, royalty-free basis to BMJ Publishing Group Ltd ("BMJ") its licensees and where the relevant Journal is co-owned by BMJ to the co-owners of the Journal, to publish the Work in this journal and any other BMJ products and to exploit all rights, as set out in our licence.

The Submitting Author accepts and understands that any supply made under these terms is made by BMJ to the Submitting Author unless you are acting as an employee on behalf of your employer or a postgraduate student of an affiliated institution which is paying any applicable article publishing charge ("APC") for Open Access articles. Where the Submitting Author wishes to make the Work available on an Open Access basis (and intends to pay the relevant APC), the terms of reuse of such Open Access shall be governed by a Creative Commons licence – details of these licences and which Creative Commons licence will apply to this Work are set out in our licence referred to above.

Other than as permitted in any relevant BMJ Author's Self Archiving Policies, I confirm this Work has not been accepted for publication elsewhere, is not being considered for publication elsewhere and does not duplicate material already published. I confirm all authors consent to publication of this Work and authorise the granting of this licence.

For peer review only

Title: Public Perception of COVID-19 Management and Response in Nigeria: A Cross-Sectional Survey

Authors: Obinna O Oleribe[1], Oliver Ezechi[2], Princess Osita-Oleribe[3], Olatayo Olawepo[4], Musa A Zaidat[2], Anddy Omolabi[5], Michael Fertleman[6], Babatunde Salako[2], Simon D. Taylor-Robinson[7].

1 – Excellence and Friends Management Care Centre (EFMC), Abuja, Nigeria

2 – Nigeria Institute for Medical Research (NIMR), Yabba, Lagos, Nigeria

3 – Center for Family Health Initiative (CFHI), Kubwa, Abuja, Nigeria

4 – Georgetown Global Health Nigeria, Abuja, Nigeria

5 - Onyozar Consult, Abuja, Nigeria

6 – Cutrale Perioperative & Ageing Group, Dept of Bioengineering, Imperial College London, White City Campus, 86 Wood Lane, London W12 0BZ, UK

7 - Department of Surgery and Cancer, Imperial College London, London, W2 1NY, UK

Corresponding Author: Dr Michael Fertleman, Cutrale Perioperative & Ageing Group, Dept of Bioengineering, Imperial College London, White City Campus, 86 Wood Lane, London W12 0BZ, UK email: m.fertleman@imperial.ac.uk

Short title: Public Perspective of COVID-19 in Nigeria

Key words: COVID-19; Nigeria; public perception; cross-sectional survey

For peer review only - http://bmjopen.bmj.com/site/about/guidelines.xhtml

Abstract

Objectives: A study designed to assess the public perception of the response of government and its institutions to the COVID-19 pandemic in Nigeria.

Setting: Self-selecting participants throughout Nigeria completed a self-administered questionnaire through an online cross-sectional survey.

Participants: 495

Results: The majority of respondents were married (76.6%), were males (61.8%), had tertiary level education (91.0%), were public servants (36.8%), Christians (82.6%), and resident either in the Federal Capital Territory (Abuja) (49.1%) or in the South-East Region of Nigeria (36.6%). Over 95% of the respondents had heard of COVID-19 (98.8%) and knew it is a viral disease (95.4%). The government and its institutions response to the pandemic were rated as poor, with the largest rating as poor for Federal President's Office (57.5%). Communication (50.0%) and prevention messages (43.7%) received the highest perception good rating. Female respondents and those less than 40 years generally rated the governmental responses as poor.

Conclusions/Recommendations: It is recommended that as a public-private partnership approached was efficiently used to more effectively disseminate public health communication and prevention messages, the Nigerian Government should expand this collaboration to improve the quality of services provided in other areas of COVID-19 outbreak management.

Article summary

Strengths and limitations of this study

- First African study of public perception of government response to the COVID 19 pandemic

For peer review only - http://bmjopen.bmj.com/site/about/guidelines.xhtml

For peer review only

- Study was cross-sectional and questionnaire was self-administered, thereby reducing researcher-induced bias but potentially increasing selection bias.
- Study provides perspectives from different classes of Nigerians on the COVID 19 pandemic
- While providing evidence for informed decisions by national leaders and program managers, the study period was short and therefore limited
- Only individuals with social media accounts or internet access could participate in the study

For peer review only

For peer review only - http://bmjopen.bmj.com/site/about/guidelines.xhtml

Introduction

There have been fundamental changes to society with the emergence of the SARS-CoV-2 global pandemic. .[1,2,3,4] As COVID-19 has no specific antiviral drug treatment or vaccine to date, universal safety precautions (USPs) and mitigating strategies are the only common way to deal with this global health emergency[4].

As COVID-19 outbreak posed significant challenges for the public health, research and medical communities, governmental policies and processes were introduced to curtail the spread of the virus across the nations of the world[5]. This included instituting temporary restrictions on travel with dramatic reduction in the number of travelers, self distancing, self isolation, quarantine of international travellers, regular hand washing and the use of face masks[5,6,7,8,9]. These policies have their economic implications to both developed and developing nations like Nigeria[10].

In Nigeria, most of these global policies were adopted and implemented without a review of their effectiveness and implications to the sociocultural climate of the nation. For instance, physical distancing measures may save many lives in high-income countries, but are less effective in poor countries with younger populations who are less susceptible to COVID-19 [11]. Such measures are associated with healthcare systemic, economic, and sociocultural challenges[10].

People living in poverty are less willing to make economic sacrifices, not because they place greater value on their livelihood concerns than contracting COVID-19[10], but because they lack the resources and social protections to isolate themselves and sacrifice economic opportunities[10]. The country formulated new policies, developed new guidelines and standard operating procedures (SOPs), and established several committees including the National Emergency Organizing Committee and Presidential Task Force (PTF) [12,12,13] to respond to the pandemic. The governmental efforts at all levels were complemented by donations and contributions from the organised private sector, health professional associations, non-governmental organisations (NGOs) and individual volunteers [14,15,16].

For peer review only

For peer review only - http://bmjopen.bmj.com/site/about/guidelines.xhtml

However, the pandemic in Nigeria continues unabated despite the various efforts of individuals, government and organised private sector with 46,803 tested, 8,344 confirmed cases and 249 deaths in 3 months (February 27 – May 27, 2020) of the pandemic in Nigeria[12], prompting plans to introduce addition measures.

The Nigerian President established the Presidential Task Force (PTF) on COVID 19 to manage the outbreak of disease across the country. The PTF was mandated to work with the Federal Ministry of Health (FMOH) and the Nigerian Center for Disease Control (NCDC) in the implementation of the various initiatives and strategies on COVID 19 containment. While FMOH and NCDC were responsible for the implementation of strategies to track the epidemic, to develop management guidelines and to engage and train field workers, the PTF was responsible for local and international relationships, mobilisation and management of resources, together with weekly briefings of the President and the Federal Nigerian Government. State and local governments also established similar COVID 19 structures that were all answerable to the local State governments of the Nigerian Federation.

The public perception, opinion and attitude to the various social control interventions introduced in the wake of the pandemic have not previously been evaluated in Africa, although some work has been produced around public perception in Europe [17] and the US [18]. Information obtained may be useful in planning, modifying and implementing a coordinated response to current and future epidemics in Nigeria. Since it is established that public perception and attitude are important for programme success, this study was designed to assess, document, and evaluate public knowledge and perception of the measures introduced to combat the COVID-19 pandemic in Nigeria.

For peer review only - http://bmjopen.bmj.com/site/about/guidelines.xhtml

Method

Timeline

This study was conducted between May 15, 2020 to May 21, 2020 inclusive, through a nationwide, anonymous online cross-sectional survey.

Participants

Participants were obtained using a self-selecting sampling technique. The participants were informed of the anonymity of their participation and that information collected would be kept confidential. Only participants 18 years and above were invited to participate in the survey

Sample size

We calculated an approximate minimum sample size of 452 at 5% precision, 95% confidence interval, and 50% response distribution (online survey) (http://www.raosoft.com/samplesize.html).

Patient and public involvement

Between 10-12 May 2020, the tool was developed. Face validity was established by trialling the survey on different members of staff from the first author's institution. Between 13-15 May 2020, the tool was reviewed with seven members of the public at the lead author's institution, before finalising with their input. A small test pilot was carried out and questions were then refined by the first authors. Further validation was limited, owing to the timeframe of the study.

No patients were involved in the design of the study.

Survey

Google Form (https://docs.google.com/forms/d/1P36vWQ0Ihf8HMTLHWIF83aZy-hFKVla7FSSm-WZudMY/edit) was used to develop and distribute the questionnaire. Between 10-12 May 2020 the tool was developed. Between 13-15 May 2020 the tool was reviewed with seven members of the public, before finalising with their input. The study was designed as an online survey to cover the entire country during the 7-day period. The questionnaire was in English and was pretested for comprehensibility,

For peer review only - http://bmjopen.bmj.com/site/about/guidelines.xhtml

acceptability and accuracy before the study was commenced. Invitations to participate in the study were through private messages, social groups, and on several social networking sites and platform (emails, WhatsApp and Facebook). By clicking on a link, the participant was directed to the survey's entry page (https://docs.google.com/forms/d/e/1FAIpQLSf4iuWGfO4pIvSVi24Sn0DhZSO9q_wnpYhB2J3boAsQ7W4Uww/viewform?vc=0&c=0&w=1&usp=mail_form_link), which contained information on the objectives of the survey, terms of participation and data privacy. Participants were also informed about the possible risks and benefits of the survey. They were asked to complete the survey in one session and that it would take between 10 to 15 minutes. Participants were able to access the survey and complete it on a computer or a mobile device.

The survey consisted of 30 semi-structured questions. The questionnaire collected information on sociodemographic characteristics, knowledge of COVID-19 and individual perspective on management and response to the pandemic. The questions were semi-structured as some questions allowed participants to provide detailed written answers to obtain a more profound sense of their perspective. The data collected for the survey and utilized in this study were stored electronically and were password protected.

Measures

Sociodemographic Characteristics: Participants responded to closed-ended demographic questions including their gender, age, relationship status, city and state of residence, work status, and educational status.

Response to COVID-19: Participant perception of the measures introduced by the Federal Government and governmental institutions to COVID-19 pandemic were assessed in the survey. The respondent perception of scores ranging from lowest score of 1 (not satisfied at all) to highest score of 10 (very satisfied) were further classified as poor (score of 1-4, average (score of 5-7) and good (scores of 8-10).

For peer review only - http://bmjopen.bmj.com/site/about/guidelines.xhtml

Data analysis

Data collected on Google Analytic Tool were exported to and analysed with the Statistical Package for Social Sciences for Windows version 23.0 (SPSS Inc., Chicago, IL). A p-value of <0.05 was considered statistically significant.

For peer review only

For peer review only - http://bmjopen.bmj.com/site/about/guidelines.xhtml

Results:

A total of 495 respondents returned completed questionnaires during the study period. While 482 (97.4%) respondents were resident in Nigeria, 13 (2.6%) were temporarily resident outside the country owing to border closures. The age of the respondents ranged from 18 to 59 years, with an average age of 42.1 years (standard deviation = 9.7). The majority of respondents were married (76.6%), males (61.8%), had tertiary level education (91.0%), were public servants (36.8%), Christians (82.6%), and resident either in the Federal Capital Territory (49.1%) or the South-East region (36.6%) (Table 1). Over 95% of the respondents had heard of COVID-19 (n = 489, 98.8%), and knew that it is a viral disease (n = 472; 95.4%).

Table 2 shows the respondent perception of the response of the Federal Government and the governmental institutions to the COVID-19 outbreak in Nigeria. The government measures were rated as poor, with the Office of the Presidency having the highest poor rating score by 57.5% of the respondents. The Federal Ministry of Health, the Presidential COVID-19 Task Force, and the Nigerian Centre for Diseases Control responses to the outbreak were rated as poor by 46.1%, 45.8% and 36.0% respectively. The respondents perception of specific governmental and institutional responses to COVID-19 are detailed in Table 3. Among the specific responses by Government and its institutions to the pandemic, public health communication about the pandemic and general prevention messages received the highest good rating by 50.0% and 43.7% of the respondents respectively. The availability of social and economic support from the Federal Government (n = 406; 82.9%) had the highest poor rating score, followed by governmental management of the resources provided for the outbreak (n = 368; 75.4%) and readiness of the government to manage the pandemic (65.5%). Social and economic support from religious bodies and family and friends were rated as poor by 52.1% and 33.1% of the respondents respectively.

The respondent perception of governmental and institutional response by respondent age group and sex is shown in table 4. The proportion of respondents less than 40 years that rated the governmental and institutional measures to combat COVID-19 as poor were greater than those 40 years and above across all aspects of the survey.

For peer review only - http://bmjopen.bmj.com/site/about/guidelines.xhtml

However, it is only in the perception of the Office of the Presidency that the difference was statistically significantly higher (p=0.03). The proportion of female respondents who rated the governmental measures as poor were higher compared to their male counterpart across all aspects of the survey. The observed difference was only statistically significantly higher again in the perception of the response of the Office of the Presidency (p=0.05).

A spatial dimension of the responses was also analysed by aggregating the ratings of the respondents by their state of residence with the results presented in figure 1. Responses were from 33 states including the Federal Capital Territory (n=33). Respondents from 10 states (30%) rated the response of government as good, while respondents from 12 states (36%) rated the governmental response as average, while the respondents from the remaining 11 states (33%) rated the responses as poor. Analyses of the rating by states, indicated that Lagos states, even though it has the highest number of laboratory confirmed COVID-19 cases in the country 3,756 (45%), rated the government response as good. This contrasts with Kano state, which comes second with 923 (11%) confirmed cases, but rated the governmental responses as poor. The FCT, which comes third in terms of confirmed cases 519 (6%), rated the government response as average. To determine the statistical significance of the relationship between the responses and the number of confirmed COVID-19 cases, the Pearson's correlation was applied. A Pearson's coefficient of 0.11 and a p value of 0.51 were obtained, indicating a weak and insignificant relationship between the responses and number of confirmed COVID-19 cases.

For peer review only - http://bmjopen.bmj.com/site/about/guidelines.xhtml

Discussion:

The success and sustainability of public health programmes are highly dependent on the positive perception and acceptance by the general public. Risk communication associated with a particular hazard involves the exchange of information and advice between experts and the public, as it becomes available. The ultimate aim of risk communication is to permit people at risk to take informed decisions to protect themselves [19]. The continued increase in new cases of COVID-19 3 months after the first case was reported in Nigeria in February 27 2020 has made Nigerians wonder about the competency of the government response to the pandemic. . As a result of this failure to cap the outbreak, significant numbers of the public are disillusioned and are not keeping to the government-issued guidelines and recommendations, with some believing that the COVID-19 pandemic is a hoax. This study was conducted to evaluate the perceptions of Nigerians to the measures taken by the Federal Government and its governmental institutions in response to the COVID-19 outbreak in the country.

In this study, the majority of the respondents were resident in Nigeria, had tertiary level education, were males having heard of COVID-19 and knew its viral aetiology (95.4%). The study shows that the respondents were educated and had some level of knowledge of COVID-19, and thus could form a meaningful opinion on the governmental response. The respondents' residency in Nigeria highlights that their opinions are well informed and could also impact on how the government guidelines and recommendations might be observed by the general public. The high educational attainment of the respondents may be a reflection of their awareness of importance and the understanding of the relevance of research. The male preponderance in this study may be a reflection of previously observation phenomena that gender plays a role in participation in online surveys[20].

The study shows that the respondents were unhappy with the manner of responses provided by the Federal Government and its agencies including the Office of the Presidency, the Presidential Task Force on COVID 19 (PTF), the Federal Ministry of Health (FMOH), and the Nigerian Center for Disease Control (NCDC). Apart from NCDC, over 70% of all respondents scored the Office of the Presidency, PTF and

For peer review only - http://bmjopen.bmj.com/site/about/guidelines.xhtml

FMOH poor or average with 57% scoring the Office of the Presidency as poor in its performance. This may be an indication of either a delayed or an inadequate response to the crisis, which may have contributed to the increasing number of new cases of COVID-19 and deaths in Nigeria 3 months after the first case was reported.

Similarly, the majority of the respondents rated specific individual measures by the government and its institutions as poor. Over 50% of the respondents scored the management of isolation, and treatment/diagnostic centres as poor. This is in support of the numerous social media videos and comments against these centres across Nigeria. Communication and delivery of prevention messages accrued better scores. This specific area of public health messaging was provided through a public-private partnership between the government agencies and global system for mobile technology operators in the country..

Unfortunately, the enforcement of the implementation of the guidelines and policies are still poor. Mainly because of the negative perception of governmental measures taken, individuals have not taken the pandemic seriously. The situation is compounded by the presentation of COVID-19 in Nigeria where a significant number of cases have been asymptomatic and the case fatality rate has been low[21] making people believe that the disease is not a serious illness[22].

Most social and economic support came from family and friends, followed by the religious bodies. The majority (over 80%) believed that government social welfare provisions were poor. One cannot however, identify the root cause of this perception as funds were made available for supportive measures for sectors of the population in Nigeria. Whether the funds were not adequately released, released but diverted or poorly distributed remains to be seen. This calls for new in-depth studies to identify the root cause of this unsatisfactory aspect of governmental performance during this crisis.

Over 65% of the participants believed that the government was not prepared for the outbreak and 75% that the government poorly managed the resources provided for the outbreak. It is not therefore surprising to note that the government was asking returnees to pay for their quarantine or be allowed to suffer the consequences – which may include denial of access to the country[23].

For peer review only - http://bmjopen.bmj.com/site/about/guidelines.xhtml

Social support, enforcement of isolation policies, and provision of testing services did not go well, according to the participants. These are project management issues that the engagement of a professional project manager to operationalize the entire process would have eliminated. It is therefore important that skilled project managers are engaged in subsequent epidemics to mitigate poor project management, better finance management, improve quality of services and to ensure timely delivery of results and benefits.

Given a large proportion of the population under the age of 40 years is active on social media, it seems prudent to recommend that governmental policy for future epidemics should use this medium for communication to a growing sector of Nigerian society.

The findings of this online cross-sectional study have to be interpreted with some limitations in mind. The use of an online survey for this study may have resulted in a selection bias for respondents who are educated, internet savvy and have a social media account. It may have limited some category of respondents from participating. Respondents without formal education, without internet connection or who live in rural areas may have been disenfranchised. However, this will not invalidate the findings presented here, more especially since only those who are knowledgeable and know the specific governmental measures taken, can meaningfully assess what is being done. Finally, this is a cross-sectional study, and does not allow any inferences to be made regarding causality and temporality with respect to perception and the socio-demographic factors.

To our knowledge, this is among the first studies to examine the perception of governmental response by the public in West Africa. Information obtained here may be useful to guide future decisions especially when considering the impact of public perception on uptake of governmental guidelines and policies. Also, for the first time in the country, we have gained information on public perception of governmental responses during a global pandemic. Finally, this study utilised nationally-representative data with appropriate sample size, which strengthens the external validity and generalizability of the study.

For peer review only - http://bmjopen.bmj.com/site/about/guidelines.xhtml

Conclusions

The study shows a generally poor perception of governmental response to the COVID-19 pandemic by the public. All the specific interventions against COVID-19 were rated as poor except for public health communication and prevention messages. It is recommended that the public-private partnership approached used in this particular area should be adopted in the implementation all areas of COVID-19 outbreak policy to streamline perceived and actual inefficiencies.

Contributorship Statement

OOO developed the concept, initial tool and first draft manuscript.
OE and POO reviewed the final tool, made inputs to the original draft manuscript, and supported data analysis.
OOO, OO, MAZ and AO did the data analysis
AO did the spatial analysis.
MF, BS and STR reviewed, edited and finalized the full manuscript.
STR provided quality assurance to the project.

Competing interest statement

The authors have no competing interests .

Funding statement

SDTR was supported by a Wellcome Trust ISSF grant at Imperial College London.

For peer review only

For peer review only - http://bmjopen.bmj.com/site/about/guidelines.xhtml

Data Sharing Statement

No additional data will be available

Acknowledgements

We are grateful to the members of the Nigerian public who helped with the survey design. We acknowledge the National Emergency Outbreak Committee on COVID 19, the Presidential Task Force (PTF) and the Nigerian Center for Disease Control. We celebrate all the frontline healthcare workers who put their lives at risk to control and contain this pandemic. We thank the EFMC and HS3 Team for their excellent work during this period. MF and SDTR are grateful to the NIHR Biomedical Facility at Imperial College London for infrastructural support.

For peer review only

For peer review only - http://bmjopen.bmj.com/site/about/guidelines.xhtml

References

1. Fang Y, Zhang H, Xie J, et al. Sensitivity of Chest CT for COVID-19: Comparison to RT-PCR. *Radiology*. February 2020:200432. doi:10.1148/radiol.2020200432

2. Huang C, Wang Y, Li X, et al. Clinical features of patients infected with 2019 novel coronavirus in Wuhan, China. *Lancet*. 2020;395(10223):497-506. doi:10.1016/S0140-6736(20)30183-5

3. WHO | Pneumonia of unknown cause – China.

4. Gudi SK, Undela K, Venkataraman R, et al. Knowledge and Beliefs towards Universal Safety Precautions to flatten the curve during Novel Coronavirus Disease (nCOVID-19) Pandemic among general Public in India: Explorations from a National Perspective. *medRxiv*. 2020:2020.03.31.20047126. doi:10.1101/2020.03.31.20047126

5. Fauci AS, Lane HC, Redfield RR. Covid-19 - Navigating the uncharted. *N Engl J Med*. 2020;382(13):1268-1269. doi:10.1056/NEJMe2002387

6. World Health Organization (WHO). Rational use of personal protective equipment for coronavirus disease 2019 (COVID-19) and considerations during severe shortages. *Who*. 2020;(April):1-28.

7. WHO. Rational use of personal protective equipment for coronavirus disease 2019 (COVID-19). *Who*. 2020;2019(February):1-7.

8. van Bavel JJ, Baicker K, Boggio PS, et al. COVID-19 pandemic response. *Nat Hum Behav*. 2020;4(5):1-12. doi:10.1038/s41562-020-0884-z

9. Raju E, Ayeb-Karlsson S. COVID-19: How do you self-isolate in a refugee camp? *Int J Public Health*. May 2020:1-3. doi:10.1007/s00038-020-01381-8

10. Barnett-Howell Z, Mobarak AM. The Benefits and Costs of Social Distancing in Rich and Poor Countries. April 2020.

11. Rubini L. Can Social Distancing Work in Low Income Countries? *SSRN Electron J*. 2020. doi:10.2139/ssrn.3611773

For peer review only - http://bmjopen.bmj.com/site/about/guidelines.xhtml

12. NCDC Coronavirus COVID-19 Microsite.

13. Presidential Task Force on Power.

14. Nigerian private sector donates more than most other African countries in fight against COVID-19 - African Business Magazine.

15. Did Nigeria Hit the 100 Billion Mark in COVID-19 Donations?

16. COVID-19: Lagos commissions 118-bed isolation centre.

17. BOL D, GIANI M, BLAIS A, LOEWEN PJ. The effect of COVID‐19 lockdowns on political support: Some good news for democracy? *Eur J Polit Res*. May 2020:1475-6765.12401. doi:10.1111/1475-6765.12401

18. Gollust SE, Nagler RH, Fowler EF. The Emergence of COVID-19 in the U.S.: A Public Health and Political Communication Crisis. *J Health Polit Policy Law*. May 2020. doi:10.1215/03616878-8641506

19. Dickmann P, Abraham T, Sarkar S, et al. Risk communication as a core public health competence in infectious disease management: Development of the ECDC training curriculum and programme. *Euro Surveill*. 2016;21(14). doi:10.2807/1560-7917.ES.2016.21.14.30188

20. Bowale A, Abayomi A, Idris J, et al. Clinical presentation, case management and outcomes for the first 32 COVID-19 patients in Nigeria. *Pamj*. 2020;35(Supp 2):1-4. doi:10.11604/PAMJ.SUPP.2020.35.2.23262

21. Smith G, Smith WG. *Does Gender Influence Online Survey Participation?: A Record-Linkage Analysis of University Faculty Online Survey Response Behavior*.; 2008.

22. Unicef warns lockdown could kill more than Covid-19 as model predicts 1.2 million child deaths.

23. Covid-19: Nigerian evacuees from Thailand must pay N297,600 for quarantine, feeding -FG - Businessday NG.

For peer review only

For peer review only - http://bmjopen.bmj.com/site/about/guidelines.xhtml

Figure 1: Spatial distribution of respondents to COVID-19 response and management in Nigeria

For peer review only

For peer review only - http://bmjopen.bmj.com/site/about/guidelines.xhtml

Table 1: Sociodemographic characteristics of respondents in the study

Characteristics	Number of respondents (%)
**Country of Residence**	
Nigeria	482(97.4)
Others	13(2.6)
**Geopolitical zone of Residence**	
**North-Central**	243(49.1)
**North-East**	29(5.9)
**North-West**	30(6.1)
**South-East**	68(13.7)
**South-South**	39(7.9)
**South-West**	86(17.4)
**Marital Status**	
Single	100(20.2)
Married	379(76.6)
Divorced/Separated	9(1.8)
Widowed	7(1.4)
**Age (years)**	
Less than 20	1(0.2)
20 - 29	61(12.3)
30 - 39	142(28.7)
40 - 49	159(32.1)
50 - 59	93(18.8)
60 years and above	39(7.9)
**Sex**	
Female	189(38.2)
Male	306(61.8)
**Religion**	
African Traditional Religion	2(0.4)
Christianity	409(82.6)
Islam	79(16.0)
Others	3(0.6)
**Education qualification**	
Non formal	18(3.6)
Primary	2(0.4)
Secondary	20(4.0)
Tertiary	455(91.9)
**Work status**	
Working	338(87.5)
Not working	38(7.7)
Student	24(4.8)

For peer review only

For peer review only - http://bmjopen.bmj.com/site/about/guidelines.xhtml

Table 2: Respondent perception of the Governmental and Governmental Institutional response to the COVID 19 pandemic in Nigeria

Government /Institution	Respondents COVID -19 response perception rating		
	Poor n (%)	Average n (%)	Good n (%)
The Presidency (N=487)	280(57.5)	130(26.7)	77(15.8)
Presidential Task Force on COVID-19 (N=491)	225(45.8)	168(34.2)	98(20.0)
Federal Ministry of Health (FMOH) (N=490)	226(46.1)	164(33.5)	490(20.4)
Nigerian Center for Disease Control (NCDC) (N=492)	177(36.0)	157(31.9)	158(32.1)

For peer review only

For peer review only - http://bmjopen.bmj.com/site/about/guidelines.xhtml

Table 3: Respondents perception of the Governmental and Governmental Institutional specific response to COVID-19 outbreak in Nigeria

Specific response	Respondents COVID -19 response perception rating		
	Poor n (%)	Average n (%)	Good n(%)
Enforcement of Stay at home, physical distancing, face mask, and handwashing policies (N=492)	244(49.6)	141(28.7)	107(21.7)
Management of Isolation Centres (N=482)	265(55.0)	148(30.7)	69(14.3)
Management of treatment and diagnostic centres (N=477)	254(53.2)	150(31.4)	73(15.3)
Communication and information about the epidemic (N=490)	129(26.3)	116(23.7)	245(50.0)
Prevention messages from government (N=490)	146(29.8)	130(26.5)	214(43.7)
Use of Personal Protective Equipment (PPE) by healthcare workers (N=484)	232(47.9)	134(27.7)	118(24.4)
Availability of social and economic support from Government (N=490)	406(82.9)	53(10.8)	31(6.3)
Availability of social and economic support from Religious bodies (N=486)	253(52.1)	129(26.5)	104(21.4)
Availability of social and economic support from family and friends (N=487)	161(33.1)	158(32.4)	168(34.5)
The readiness of the government to manage the epidemic (N=490)	321(65.5)	102(20.8)	67(13.7)
Government management of the resources provided for the outbreak (N=488)	368(75.4)	82(16.8)	38(7.8)

For peer review only

For peer review only - http://bmjopen.bmj.com/site/about/guidelines.xhtml

Table 4: The Respondents perception of Government and Institution response to COVID 19 pandemic in Nigeria by Sex and Age-group

Government/Institution	Respondents COVID -19 response perception rating						P-value
	Poor	Average	Good	Poor	Average	Good	
	**Male**			**Female**			
**The Presidency (N=487)**	167(59.6)	80(61.5)	56(72.7	113(40.4)	50(38.5)	21(27.3	0.05
**Presidential Task Force on COVID-19 (N=491)**	130(57.8)	112(66.7)	62(63.3)	95(42.2)	56(33.3)	36(36.7)	0.19
**Federal Ministry of Health (FMOH) (N=490)**	128(56.6)	115(70.1)	60(60.0)	98(43.4)	49(29.9)	40(40.0)	0.23
**Nigerian Center for Disease Control (NCDC) (N=492)**	104(58.8)	102(65.0)	99(62.7)	73(41.2)	55(35.0)	59(37.3)	0.44
	**Less than 40 years**			**40 years and above**			
**The Presidency (N=487)**	126(45.0)	51(39.2)	25(32.5)	154(55.0)	79(60.8)	56(72.7)	0.03
**Presidential Task Force on COVID-19 (N=491)**	98(43.6)	70(41.7)	35(35.7)	127(56.4)	98(58.3)	63(64.3)	0.21
**Federal Ministry of Health (FMOH) (N=490)**	92(40.7)	73(44.5)	38(38)	134(59.3)	61(55.5)	62(62.0)	0.83
**Nigerian Center for Disease Control (NCDC) (N=492)**	68(78.4)	70(44.6)	66(41.8)	109(61.6)	87(55.4)	92(58.2)	0.51

For peer review only - http://bmjopen.bmj.com/site/about/guidelines.xhtml

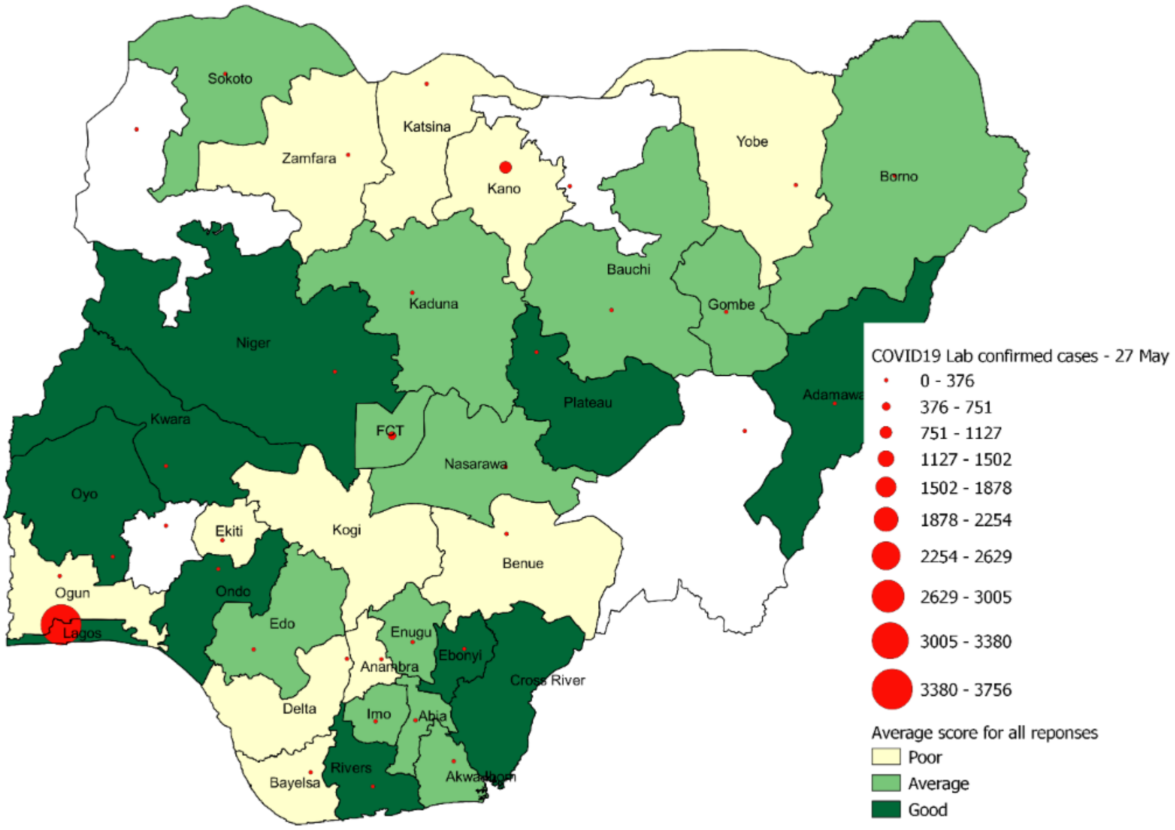
Sokoto
Zamfara
Katsina
Kano
Yobe
Borno
Bauchi
Gombe
Kaduna
Niger
Plateau
Adamawa
Kwara
FCT
Nasarawa
Oyo
Ekiti
Kogi
Benue
Ogun
Ondo
Lagos
Edo
Enugu
Ebonyi
Anambra
Cross River
Delta
Imo
Abia
Rivers
Bayelsa
Akwa Ibom
COVID19 Lab confirmed cases - 27 May
0 - 376
376 - 751
751 - 1127
1127 - 1502
1502 - 1878
1878 - 2254
2254 - 2629
2629 - 3005
3005 - 3380
3380 - 3756
Average score for all reponses
Poor
Average
Good

review only
